# Naval Board of Examiners

**Published:** 1861-03

**Authors:** 


NAVAL BOARD OF EXAMINERS.—A Naval Board of Surgeons, for the examination of Assistant Surgeons for promotion, will convene at the Naval Asylum, in this city, on the first of March next. The Board will consist of Surgeons James M. Green, J. M. Foltz, and C. H. Wheelwright, and Passed Assistant Surgeon John Y. Taylor, Recorder.

